# The highly accuracy of lymphocyte count in diagnosing influenza A in children during the influenza A season

**DOI:** 10.3389/fped.2025.1609822

**Published:** 2025-09-26

**Authors:** Houyu Chen, Penghao Cui, Haifeng Jin, Yan Guo, Yi Huang, Feng Jiao, Xiaotao Yang, Yanchun Wang, Yonghan Luo

**Affiliations:** ^1^ Second Department of Infectious Disease, Kunming Children's Hospital (Children's Hospital Affiliated to Kunming Medical University), Kunming, Yunnan, China; ^2^ Yunnan Key Specialty of Pediatric Infection (Training and Education Program)/Kunming Key Specialty of Pediatric Infection, Kunming, China; ^3^ Faculty of Life Science and Technology, Kunming University of Science and Technology, Kunming, Yunnan, China; ^4^ Department of Reproductive Gynecology, The First People’s Hospital of Yunnan Province, Kunming, China

**Keywords:** influenza A, complete blood count, lymphocyte count, diagnosis, children

## Abstract

**Objective:**

To study the value of lymphocyte count in early diagnosis of influenza A in children during the influenza A season.

**Methods:**

We selected children aged 0–18 years diagnosed with influenza A who visited the outpatient clinic of Kunming Children's Hospital during the influenza A epidemic in China from March to April 2023 as the case group (influenza group) and matched them with healthy children undergoing physical examinations as the control group. We collected complete complete blood count within 48 h of disease onset in children with influenza A and compared these parameters with those of the control group. Receiver operating characteristic (ROC) curve analysis, restricted cubic spline functions, and decision curve analysis (DCA) were employed to assess diagnostic performance and clinical utility.

**Results:**

The results showed that there were 541 children in the influenza group and 597 in the control group, with no statistically significant differences in age and gender distribution between the two groups (*P* > 0.05). Significant differences (*P* < 0.05) were observed in white blood cell count (WBC), neutrophil percentage, neutrophil count, lymphocyte percentage, lymphocyte count, monocyte percentage, monocyte count, platelet count (PLT), and C-Reactive Protein (CRP) between the two groups. ROC curve analysis indicated that lymphocyte count had the highest diagnostic accuracy for influenza A, with a Receiver-operating-characteristic curve (AUC) of 0.926 (95% CI 0.9113–0.9413) and an optimal cutoff value of 1. 812 × 10^9^.Subgroup analyses stratified by age revealed consistently high AUC values. Dose-response analysis demonstrated a significant non-linear relationship between lymphocyte count and influenza A infection risk (nonlinear test, *P* < 0.001). DCA indicated favorable clinical applicability of lymphocyte count as a predictive marker.

**Conclusion:**

During the influenza A epidemic, a decrease in lymphocyte count within 48 h of onset can serve as an indicator for early detection and diagnosis of pediatric influenza A.

## 1 Introduction

Influenza remains a significant threat to children's health (1, 2). Some studies (3) suggest that delays in diagnosis and administration of Oseltamivir are associated with increased mortality. The “gold standard” for influenza diagnosis is viral isolation and culture, which are time-consuming, technically demanding, and have limited clinical application. Other important ancillary tests include viral nucleic acid, antigen, and antibody detection. Nucleic acid testing for influenza A is highly sensitive and specific and is faster than culture, but it still takes several hours and requires technical expertise, making it challenging for some developing countries or primary care hospitals to implement (4). The rapid antigen detection test for influenza facilitates the immediate identification of viral antigens, offering the advantage of delivering results within minutes and requiring minimal procedural complexity. As a result, it is extensively utilized in outpatient settings and primary healthcare institutions. However, when compared to nucleic acid testing, its sensitivity is subject to variation and is generally lower, thereby limiting the reliability of early diagnosis (5). Consequently, in resource-constrained environments, there remains a pressing need for accessible, user-friendly, and cost-effective tools to support the early suspicion of influenza A.

Complete blood count (CBC) testing is the most commonly used laboratory examination in pediatric fever clinics. Although several studies have explored the relationship between CBC parameters and influenza virus infections, certain limitations persist. Some studies (6–8) have focused on composite indices such as the neutrophil-to-lymphocyte ratio (NLR), lymphocyte-to-monocyte ratio (LMR), and mean platelet volume to platelet count ratio (MPV/PLT) in relation to influenza. However, these ratio-based indicators are not the primary focus in routine CBC interpretation, and their diagnostic performance, often reflected in the area under the receiver operating characteristic (ROC) curve (AUC), tends to be suboptimal. Furthermore, while previous research has evaluated the accuracy of CBC in diagnosing influenza through ROC analysis, the presence of a linear dose-response relationship between relevant indicators and diagnostic probability remains unclear. More importantly, there is a lack of in-depth analysis regarding the practical clinical value of individual CBC parameters for influenza screening or diagnosis, as reflected by the net benefit in clinical decision curve analysis (DCA).

For the reasons outlined above, we employed a comprehensive approach utilizing three methodologies: ROC curve analysis (to assess diagnostic accuracy), restricted cubic spline (RCS) analysis (to explore linear/nonlinear relationships), and DCA (to evaluate clinical utility). These methods were systematically and specifically applied to assess the diagnostic value of lymphocyte count, a commonly used CBC parameter, in the diagnosis of pediatric influenza A. This study aims to provide an in-depth exploration of the diagnostic significance of commonly used CBC parameters in pediatric influenza A, with the goal of aiding early clinical detection of influenza cases and reducing the incidence of severe complications.

## 2 Materials and methods

### 2.1 Data source

We selected children aged 0–18 years diagnosed with influenza A who visited the outpatient clinic of Kunming Children's Hospital during the influenza A epidemic in China from March to April 2023. The study protocol was approved by the Ethics Review Committee of the Children's Hospital of Kunming Medical University. Due to the retrospective nature of the study, the requirement for informed consent was waived, and all patient information was anonymized.

### 2.2 Study population

The inclusion criteria were as follows: (1) Outpatients children under 18 years of age; (2) In accordance with the Expert consensus on the diagnosis and treatment of Influenza A in Children (9). Exclusion criteria: (1) co-infection with other pathogens or other sites of infection; All enrolled pediatric participants underwent standardized screening for prevalent respiratory pathogens, including respiratory syncytial virus (RSV), adenovirus, parainfluenza virus, and *mycoplasma pneumoniae*, utilizing rapid antigen assays, serological analyses, or multiplex polymerase chain reaction (PCR) panels. Subjects exhibiting confirmed co-infections, as identified through these diagnostic modalities, were systematically excluded from the investigation. In instances where concurrent bacterial infection was clinically suspected, microbiological cultures of blood or sputum specimens were conducted. (2) hematologic malignancies, immunodeficiencies, or use of immunosuppressive agents; (3) significant lack of clinical data.

The children who tested positive for Influenza A via reverse-transcriptase polymerase chain reaction (RT-PCR) were included in the case group (Influenza group), and were matched with healthy children and influenza-negative children primarily on age (±6 months). Efforts were made to balance gender distribution across groups, although strict gender matching was not applied. The healthy children underwent routine physical examinations (Healthy group), and the influenza-negative children presented with influenza-like illness but tested negative for Influenza A (Negative group), who served as the control group. Data for all groups were collected during the same influenza season (March to April 2023) to minimize temporal bias.

### 2.3 Study variables and data extraction

The white blood cell count (WBC, ×10⁹ /L), neutrophil count (NC, ×10^9^ /L), lymphocyte count (LC, ×10^9^ /L), monocyte count (MC, ×10^9^ /L), red blood cell count (RBC, ×10^12^ /L), hemoglobin (Hb, g/L), platelet count (PLT, ×10^9^ /L) and other indicators of routine blood test were collected. Influenza A virus nucleic acid was detected by “respiratory virus nucleic acid multiplex detection (PCR fluorescent probe method).

### 2.4 Statistical analysis

Statistical analyses were conducted using R software (version 4.4.1). The *χ*^2^ test and Fisher's exact test were employed to compare categorical variables, while *t*-tests or Mann–Whitney tests were used for continuous variables. The ROC curve was plotted to determine the optimal cut-off value, which was selected using the Youden index (Youden index = Sensitivity + Specificity−1). RCS functions were utilized to visually assess the association between variables and outcomes. DCA was employed to evaluate the clinical utility of the variables. Statistical significance was set at *P* < 0.05.

## 3 Results

### 3.1 Clinical features of included patients (influenza group and healthy group)

**Figure 1:**
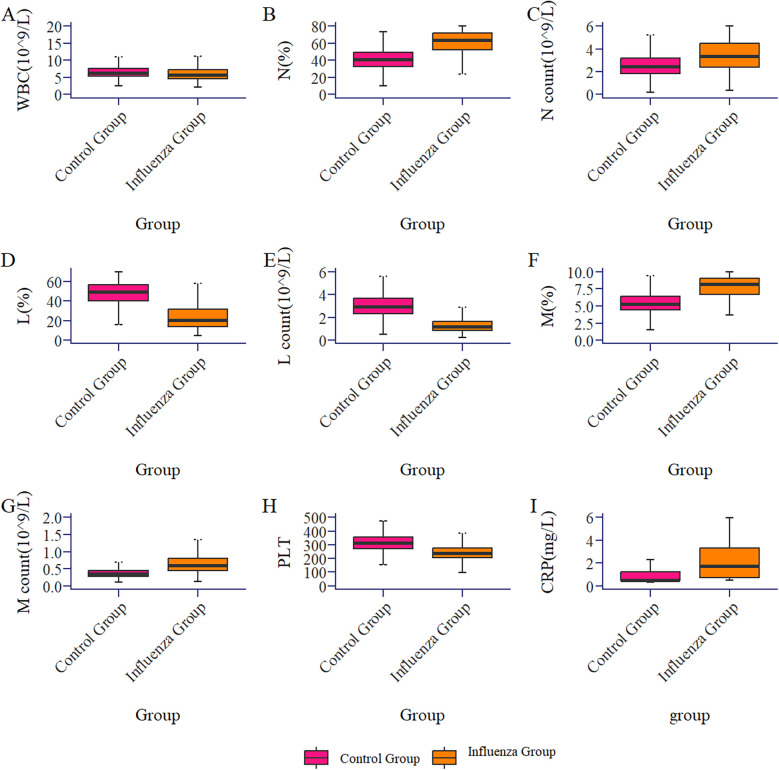
Box plot of the comparison of routine blood parameters between the influenza group and the control group (healthy group).

The results showed that there were 541 children in the influenza group and 597 in the control group, with no statistically significant differences in age and gender distribution between the two groups (*P* > 0.05). The age of influenza A group was 6.00[4.00, 8.00] years old, including 313 males (57.9%) and 228 females (42.1%). The control group was 6.00[4.00,8.00] years old, including 337 boys and children (56.4%) and 260 girls (43.6%). Significant differences (*P* < 0.05) were observed in WBC, N%, NC, L%, LC, M%, MC, PLT, and CRP between the two groups (see Table 1; Figure 1). However, there was no significant difference in M count and RBC between the two groups.

**Table 1:** Comparison of complete blood count parameters between influenza group and control group (healthy group).

Characteristics	Overall (*n* = 1,138)	Control group (*n* = 597)	Influenza group (*n* = 541)	*P*
Sex (girl), *n* (%)	488 (42.9)	260 (43.6)	228 (42.1)	0.675
Age [median (IQR)], years	6.00 [4.00, 8.00]	6.00 [4.00, 8.00]	6.00 [4.00, 8.00]	0.443
WBC [median (IQR)], ×10^9^ /L	6.09 [4.98, 7.54]	6.26 [5.34, 7.63]	5.80 [4.66, 7.44]	<0.001
N%[median (IQR)], %	52.40 [38.60, 68.70]	40.70 [33.00, 49.70]	68.60 [57.70, 76.30]	<0.001
N count [median (IQR)], ×10^9^ /L	3.06 [2.15, 4.50]	2.47 [1.86, 3.34]	3.97 [2.75, 5.26]	<0.001
L% [median (IQR)], %	36.95 [19.40, 51.30]	50.10 [41.50, 58.50]	19.30 [13.50, 28.60]	<0.001
L count [median (IQR)], ×10^9^ /L	2.10 [1.13, 3.11]	2.99 [2.37, 3.76]	1.11 [0.79, 1.54]	<0.001
M% [median (IQR)], %	7.35 [5.20, 10.60]	5.40 [4.40, 6.60]	10.70 [8.50, 13.40]	<0.001
M count [median (IQR)], ×10^9^ /L	0.44 [0.32, 0.64]	0.35 [0.28, 0.44]	0.62 [0.46, 0.83]	<0.001
RBC [median (IQR)], ×10^12^ /L	4.91 [4.67, 5.14]	4.89 [4.66, 5.12]	4.93 [4.69, 5.15]	0.214
Hb [median (IQR)], g/L	137.00 [130.00, 144.00]	137.00 [130.00, 144.00]	137.00 [131.00, 143.00]	0.686
PLT [median (IQR)], ×10^9^ /L	276.50 [229.00, 329.75]	313.00 [271.00, 360.00]	239.00 [208.00, 280.00]	<0.001
CRP [median (IQR)], mg/L	1.87 [0.59, 4.64]	0.50 [0.50, 1.32]	2.74 [1.11, 5.75]	<0.001

IQR, interquartile range; WBC, white blood cell; Hb, hemoglobin; PLT, platelet; N%, percentage of neutrophils; M%, percentage of monocyte; M count, monocyte count; N count, Neutrophil count; L%, percentage of lymphocytes; L count, lymphocyte count; CRP, C -reactive protein. All variables, except Sex, were analyzed using the Mann–Whitney *U* test, while Sex was analyzed with the chi-square test.

### 3.2 Diagnostic value of lymphocyte count for influenza infection in children (influenza A group and healthy group)

**Figure 2:**
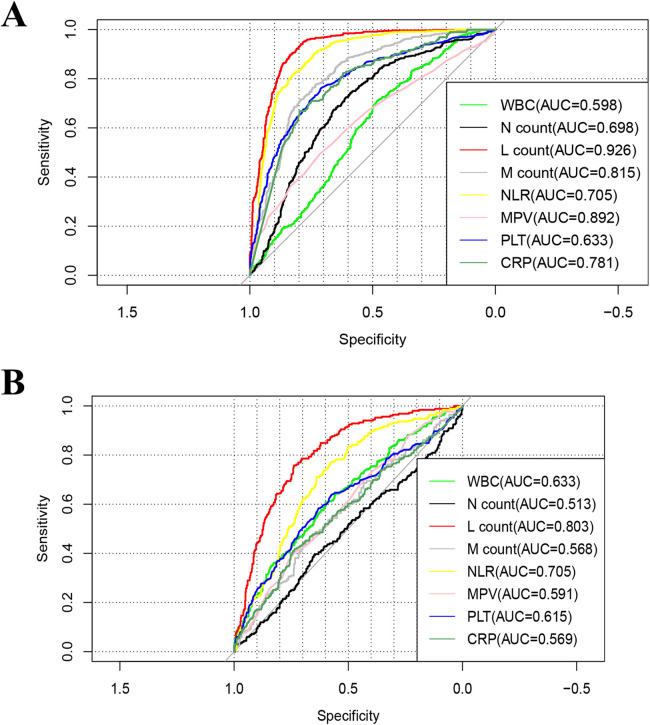
Receiver-operating-characteristic (ROC) curve of the of complete blood countparameters for influenza A. **(A)** Influenza group vs. Healthy group, **(B)** influenza group VS Negative group. WBC, white blood cell; Hb, hemoglobin; PLT, platelet; N%, percentage of neutrophils; M%, percentage of monocyte; M count, monocyte count; N count, neutrophil count; L%, percentage of lymphocytes; L count, lymphocyte count; CRP, C -reactive protein.

ROC curve analysis (Figure 2A) indicated that LC had the highest diagnostic accuracy for influenza A, with an AUC of 0.926 (95% CI 0.911–0.941) and an optimal cutoff value of 1.812 × 10^9^/L. Considering the differences in normal LC across age groups, we conducted separate analyses for different age groups.

The AUC for children under 1 year of age was 0.831 (0.724–0.937), with an optimal diagnostic threshold of 3.586 × 10^9^/L. For children 2–3 years old, the LC AUC was 0.951(95% CI 0.921–0.981) with an optimal diagnostic cutoff of 2.239 × 10^9^/L; for those aged 4–6 years, the AUC was 0.943(95% CI 0.924–0.963) with an optimal cutoff of 1.97 × 10^9^/L; and for those ≥7 years old, the AUC was 0.949(95% CI 0.930–0.967) with an optimal cutoff of 1.703 × 10^9^/L (see Table 3).

**Table 3:** The highly accuracy of lymphocyte count in diagnosing influenza A.

Comparison group	Age group	ROC AUC (95% CI)	Cut-off	Sensitivity (95% CI)	Specificity (95% CI)	PPV (95% CI)	NPV (95% CI)	LR+	LR−
Influenza A vs. Healthy	<1 year	0.831 (0.724–0.937)	3.586	0.793 (0.693–0.893)	0.824 (0.729–0.918)	0.793 (0.693–0.893)	0.824 (0.729–0.918)	4.494	0.251
	2-3 years	0.951 (0.921–0.981)	2.239	0.821 (0.756–0.885)	0.917 (0.871–0.963)	0.928 (0.884–0.971)	0.797 (0.730–0.864)	9.846	0.196
	4–6 years	0.943 (0.924–0.963)	1.97	0.862 (0.832–0.891)	0.921 (0.898–0.944)	0.927 (0.905–0.950)	0.850 (0.819–0.881)	10.885	0.150
	≥7 years	0.949 (0.930–0.967)	1.703	0.852 (0.820–0.883)	0.938 (0.917–0.960)	0.940 (0.918–0.961)	0.848 (0.816–0.880)	13.747	0.158
Influenza A vs. Negative	<1 year	0.672 (0.501–0.843)	1.891	0.452 (0.312–0.591)	0.889 (0.801–0.977)	0.875 (0.782–0.968)	0.485 (0.345–0.625)	4.065	0.617
	2–3 years	0.823 (0.725–0.920)	2.117	0.742 (0.650–0.834)	0.857 (0.784–0.931)	0.742 (0.650–0.834)	0.857 (0.784–0.931)	5.194	0.301
	4–6 years	0.833 (0.787–0.878)	1.456	0.600 (0.548–0.652)	0.890 (0.857–0.924)	0.806 (0.763–0.848)	0.746 (0.699–0.792)	5.457	0.449
	≥7 years	0.809 (0.758–0.860)	1.109	0.494 (0.440–0.549)	0.943 (0.918–0.969)	0.918 (0.887–0.948)	0.594 (0.540–0.647)	8.715	0.536

ROC AUC, receiver operating characteristic area under the curve; CI, confidence interval; PPV, positive predictive value; NPV, negative predictive value; LR+, positive likelihood ratio; LR−, negative likelihood ratio.

### 3.3 Dose-response analysis of lymphocyte count for influenza A infection in children (influenza A group and healthy group)

**Figure 3:**
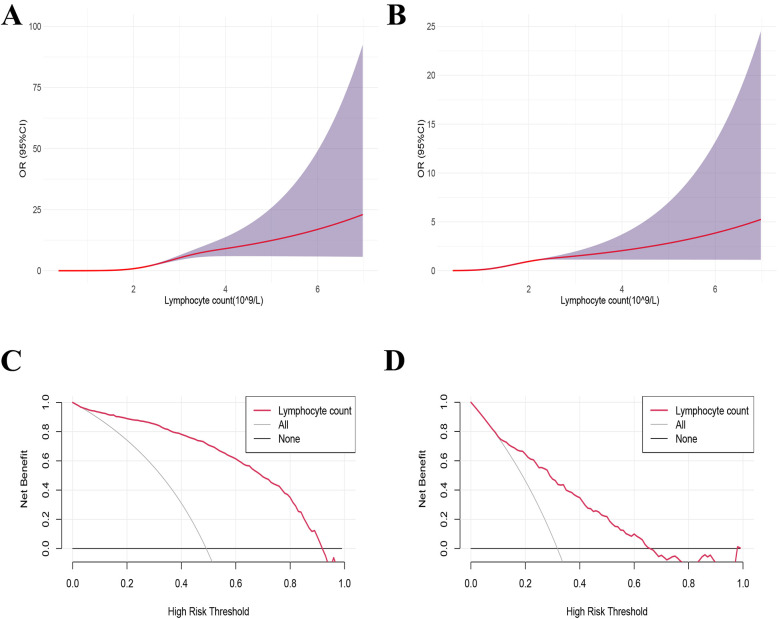
Dose-response relationship and decision curve analysis between lymphocyte counts and the influenza A. **(B)** Decision curve analysis (DCA) of lymphocyte counts for influenza A. **(A,C)** Influenza group VS Healthy group, **(B,D)** influenza group VS Negative group.

Using 1.812 × 10^9^ /L as the reference value, the dose-response relationship between LC and influenza A infection was analyzed using the restricted cubic spline method combined with spline functions and logistic regression. In Figure 3A, the *x*-axis represents the continuous change in LC, and the *y*-axis represents the corresponding predicted odds ratio (OR). The shaded area indicates the 95% confidence interval. There was a nonlinear dose-response relationship between LC and influenza A infection (nonlinear test, *P* < 0.001).

### 3.4 Decision curve analysis of lymphocyte count and influenza A infection in children (influenza A group and healthy group)

The decision curve was plotted with the net benefit on the *y*-axis and the high-risk threshold on the *x*-axis, with the medium-high risk threshold set between 0 and 1. Figure 3C demonstrated that when the high-risk threshold ranged from 0.2 to 1, the net benefit was greater than 0. This indicated that using LC to predict the prognosis of influenza A provides a net clinical benefit, particularly in identifying patients at higher risk of severe outcomes. By setting the threshold within this range, LC exhibits potential as a valuable predictive factor for clinical decision-making, aiding clinicians in identifying patients who may benefit from more intensive monitoring or treatment.

### 3.5 Comparison of clinical characteristics between influenza A group and negative group

This study included 541 cases in the Influenza group and 252 cases in the negative group, with no statistically significant differences in age and gender distribution between the two groups (*P* > 0.05). There were statistically significant differences in WBC, N%, L%, LC, RBC, M%, MC, Hb, PLT, and CRP between the two groups (*P* < 0.05) (Table 2).

**Table 2:** Comparison of complete blood countparameters between influenza group and negative group).

Characteristics	Overall (*n* = 793)	Influenza group (*n* = 252)	Negative group (*n* = 541)	*P*
Sex (girl), *n* (%)	350 (44.1)	228 (42.1)	122 (48.4)	0.114
Age [median (IQR)], years	6.00 [4.00, 8.00]	6.00 [4.00, 8.00]	5.00 [4.00, 7.25]	0.052
WBC [median (IQR)], ×10^9^/L	6.10 [4.85, 7.92]	5.80 [4.66, 7.44]	6.89 [5.34, 8.79]	<0.001
N% [median (IQR)], %	65.00 [54.60, 73.93]	68.60 [57.70, 76.30]	58.76 [49.82, 66.98]	<0.001
N count [median (IQR)], ×10^9^/L	3.95 [2.75, 5.43]	3.97 [2.75, 5.26]	3.92 [2.77, 5.78]	0.57
L% [median (IQR)], %	22.75 [15.30, 32.44]	19.30 [13.50, 28.60]	29.39 [22.04, 37.87]	<0.001
L count [median (IQR)], ×10^9^/L	1.32 [0.93, 1.95]	1.11 [0.79, 1.54]	1.97 [1.50, 2.63]	<0.001
M% [median (IQR)], %	10.40 [8.30, 13.10]	10.70 [8.50, 13.40]	9.88 [8.01, 12.36]	0.002
M count [median (IQR)], ×10^9^/L	0.64 [0.47, 0.85]	0.62 [0.46, 0.83]	0.70 [0.52, 0.90]	0.002
RBC [median (IQR)], ×10^12^/L	4.90 [4.65, 5.12]	4.93 [4.69, 5.15]	4.82 [4.61, 5.04]	<0.001
Hb [median (IQR)], g/L	137.00 [130.00, 143.00]	137.00 [131.00, 143.00]	135.50 [129.00, 142.00]	0.002
PLT [median (IQR)], ×10^9^/L	247.00 [211.00, 293.00]	239.00 [208.00, 280.00]	269.00 [223.00, 317.25]	<0.001
CRP [median (IQR)], mg/L	3.04 [1.17, 6.73]	2.74 [1.11, 5.75]	3.86 [1.53, 8.77]	0.002

IQR, interquartile range; WBC, white blood cell; Hb, hemoglobin; PLT, platelet; N%, percentage of neutrophils; M%, percentage of monocyte; M count, Monocyte count; N count, neutrophil count; L%, percentage of lymphocytes; L count, lymphocyte count; CRP, C -reactive protein. All variables, except Sex, were analyzed using the Mann–Whitney *U* test, while Sex was analyzed with the chi-square test.

ROC curve analysis (Figure 2B) showed that LC had the highest diagnostic accuracy for Influenza A, with an AUC of 0.803 (0.770–0.834) and an optimal cutoff value of 1.479 × 10^9^/L. Considering the variations in normal LC levels across different age groups, separate analyses were conducted for each group. For children under 1 year of age, the AUC was 0.672 (0.501–0.843), with an optimal diagnostic threshold of 1.891 × 10^9^/L; for children aged 2–3 years, the AUC was 0.823 (95% CI 0.725–0.920), with an optimal diagnostic threshold of 2.117 × 10^9^/L; for the 4–6 year group, the AUC was 0.833 (95% CI 0.787–0.877), with an optimal cutoff value of 1.456 × 10^9^/L; and for the ≥7 years group, the AUC was 0.809 (95% CI 0.758–0.859), with an optimal threshold of 1.109 × 10^9^/L (see Table 3).

Using 1.479 × 10^9^/L as a reference value, a RCS method combined with spline functions and logistic regression was applied to analyze the dose-response relationship between LC levels and Influenza A infection. The results revealed a non-linear dose-response relationship between LC and Influenza A infection (non-linearity test, *P* < 0.001) (Figure 3B). Figure 3D indicates that, when the high-risk threshold ranges from 0.1 to 0.7, the net benefit exceeds zero, suggesting that the use of LC to predict the prognosis of Influenza A provides a net clinical benefit.

## 4 Discussion

In this study, we conducted a retrospective analysis to explore the diagnostic value of LC during an influenza A outbreak in children. Our findings indicate that LC possesses high diagnostic accuracy for pediatric influenza A during flu seasons. By utilizing this fundamental and straightforward marker, clinicians can promptly assess the likelihood of influenza infection in children. This research aids healthcare professionals in the early identification and timely treatment of influenza.

Influenza A is an acute respiratory infectious disease caused by the influenza A virus, characterized by high fever, with temperatures reaching 39–40° C, accompanied by chills, sore throat, nasal congestion, and coughing (10). It may also present with gastrointestinal symptoms such as vomiting and abdominal pain. Patients typically experience pronounced systemic symptoms, including fatigue, generalized muscle aches, and loss of appetite (11). In some cases, the illness can progress to severe influenza, making early detection and active treatment particularly crucial for children under five years old, especially those under two. A complete blood count is a simple, rapid, and effective laboratory tool for assessing infectious inflammation; it provides timely insights into the pathophysiological changes of the disease, with certain parameters reflecting the immune status of the body. Lymphocytes are the primary immune cells responsible for viral eradication, and in virus-related infections, the circulating blood LC typically increases. However, this study found that within 48 hours of the onset of influenza A, the influenza group's CBC showed a significant reduction in LC compared to the control group. This mechanism may be related to lymphocyte redistribution and increased apoptosis (12, 13). After invading the human body, influenza virus-specific lymphocytes bind to the virus and migrate to inflammation sites such as the lungs and secondary lymphoid organs. These recruited cells play a crucial role in defending against viral infections, leading to lymphocyte depletion and subsequent reduction in peripheral blood LC. Studies have found infiltrative apoptotic lymphocytes in the lungs of fatal influenza A patients, and uninfected lymphocyte apoptosis is considered a key mechanism of virus-induced lymphocyte depletion (14).

This study found that although children with influenza A exhibited significant differences from healthy controls across multiple CBC parameters, ROC curve analysis revealed that the diagnostic performance (AUC values) of WBC, NC, and C- CRP was relatively low. As a composite reflection of multilineage changes, WBC levels may be elevated or decreased in a variety of infectious diseases (viral or bacterial) and even autoimmune conditions, thereby lacking sufficient specificity (15). Although the absolute NC may show a transient increase during the early phase of infection due to acute inflammatory responses, this change reflects nonspecific inflammation and, according to traditional clinical understanding, is more commonly associated with bacterial infections. Consequently, its diagnostic amplitude and stability are inferior to the characteristic decline observed in LC (16). Similarly, CRP, as a broad-spectrum inflammatory marker, primarily indicates a nonspecific systemic inflammatory state and lacks specificity in distinguishing viral etiologies (17). Therefore, during seasonal outbreaks of influenza A, the LC within 48 h of onset demonstrates superior diagnostic performance owing to its ability to more directly, sensitively, and specifically reflect lymphotropic damage caused by influenza A virus infection.

This study also has several limitations. First, as a single-center retrospective study, it is inevitably subject to selection bias. The study population primarily comprised children with mild outpatient influenza A, which may not fully represent the broader pediatric influenza A population, thereby limiting the generalizability of the findings. Future research should consider including patients with symptom onset beyond 48 hours, as well as those with moderate to severe disease, to validate the diagnostic performance of lymphocyte count across different stages and severities of illness. Moreover, future investigations could explore the integration of LC with other biomarkers, such as cytokines, to enhance diagnostic accuracy and better capture the full clinical course of influenza A.

## 5 Conclusion

During the influenza A epidemic, a decrease in LC within 48 h of onset can serve as an indicator for early detection and diagnosis of pediatric influenza A.

## Data Availability

The raw data supporting the conclusions of this article will be made available by the authors, without undue reservation.
